# Trigger Point Therapy Techniques as an Effective Unconventional Method of Treating Tension Headaches: A Systematic Review

**DOI:** 10.3390/healthcare12181868

**Published:** 2024-09-17

**Authors:** Aleksandra Dolina, Michał Baszczowski, Wiktor Wilkowicz, Grzegorz Zieliński, Jacek Szkutnik, Piotr Gawda

**Affiliations:** 1 Interdisciplinary Scientific Group of Sports Medicine, Department of Sports Medicine, Medical University of Lublin, 20-093 Lublin, Poland; aleksandra.dolina.um@gmail.com (A.D.); m.baszczowski@gmail.com (M.B.); wiktor.wilkowicz@wp.pl (W.W.); 2 Department of Sports Medicine, Medical University of Lublin, 20-093 Lublin, Poland; piotr.gawda@umlub.pl; 3 Independent Unit of Functional Masticatory Disorders, Medical University of Lublin, 20-093 Lublin, Poland; jacek.szkutnik@umlub.pl

**Keywords:** tension-type headaches, trigger point, physiotherapy, primary headaches

## Abstract

**Background/Objectives** The main aim of the literature review was to determine whether different trigger point therapy techniques are effective in decreasing the intensity, frequency, and duration of tension-type headaches. An additional aim was to assess the impact of trigger point therapy on other physical and psychological variables in tension-type headaches. **Methods** This literature review was conducted in accordance with PRISMA guidelines, and the inclusion and exclusion criteria were developed using the PICO(s) strategy. Searches were carried out in four databases: PubMed, Science Direct, Cochrane Library, and PEDro. **Results** Of the 9 included studies with 370 participants, 6 studies were randomised controlled trials, 2 were pilot studies, and 1 was a case report. **Conclusions** Trigger point therapy has reduced the duration, intensity, and frequency of headaches. Dry needling, ischaemic compression, Positional Relaxation Techniques, and massage protocols focused on deactivating trigger points are effective methods of unconventional treatment of tension-type headaches.

## 1. Introduction

Tension-type headaches (TTHs) are defined as bilateral pressing or tightening types of pain with mild to moderate intensity. The intensity of pain is not increased by physical activity, nor is it associated with vomiting and nausea [1]. TTHs are considered the most common type of primary headaches across all age categories [2]. According to the 2013 Global Burden of Disease Study, the prevalence of TTHs is estimated at 21.75% of the population, regardless of age, and is considered the second most common chronic disease worldwide [3]. This condition is regarded as a significant socio-economic problem, accounting for around 9% of annual sick leave. TTHs are also accompanied by a reduction in the quality of life and have a negative impact on the degree of disability [4]. In many cases, TTHs accompany such disorders as fibromyalgia, anxiety, depression, sleep disturbances, or hypothyroidism [5]. However, despite the significant impact of tension-type headaches on public health, the aetiology and mechanism of this condition are not fully established. It has been suggested that peripheral factors are associated with episodic tension-type headaches, while central factors are involved in the development of chronic pain [6]. Another important factor involved in tension-type headaches is muscle dysfunction, including myofascial trigger points [7]. 

Myofascial trigger points (MTrPs) are defined as small, hypersensitive areas within muscle fibres with a palpable lump and tissue thickening. Under pressure, they cause tenderness and pain that is referred to another region, sometimes distant from the MTrPs. Additionally, it is often accompanied by a local twitch response, i.e., a contraction of individual fibres (not the entire muscle) [8]. The causes of trigger points are not entirely known; however, the most likely Integrated Trigger Point Hypothesis (ITPH) states that many factors affect the sarcomeres and motor endplates, making them hyperactive. This, in turn, causes pathological changes at the cellular level, including reduced oxygen supply, involuntarily shortened muscle fibres, abnormal biochemical composition with elevated concentrations of acetylcholine, noradrenaline, and serotonin, and a lower pH [9,10]. These findings could be linked to the peripheral and central sensitisation model which helps in understanding chronic or amplified pain [9,11]. In addition, trigger point pain could project to the head and neck areas, reproducing the pain pattern of tension-type headaches [12].

TTH treatment relies on both conventional and unconventional methods. One of the most commonly used non-conventional methods is manual therapy (MT) [13]. It has been proven that the use of MT in the treatment of TTH can be more effective than usual General Practise care [14]. However, there has been no literature review focused solely on the use of myofascial trigger point therapy in the treatment of TTH so far. Therefore, the main aim of the literature review was to determine whether different trigger point therapy techniques are effective in decreasing the intensity, frequency, and duration of tension-type headaches. An additional aim was to assess the impact of trigger point therapy on other physical and psychological variables in tension-type headaches.

The research questions for this systematic review were:

1. Are various trigger points therapy techniques effective in treatment of tension-type headaches?
2. How trigger points therapy affect physical and psychological variables in tension-type headaches?

## 2. Materials and Methods

This literature review was conducted according to the Preferred Reporting Items for Systematic Reviews and Meta-Analysis (PRISMA) Statement [15]. 

### 2.1. Eligibility Criteria

The inclusion and exclusion criteria presented in Table 1 were developed according to the PICO(S) strategy [16]. 

### 2.2. Search Strategy

**Figure 1:**
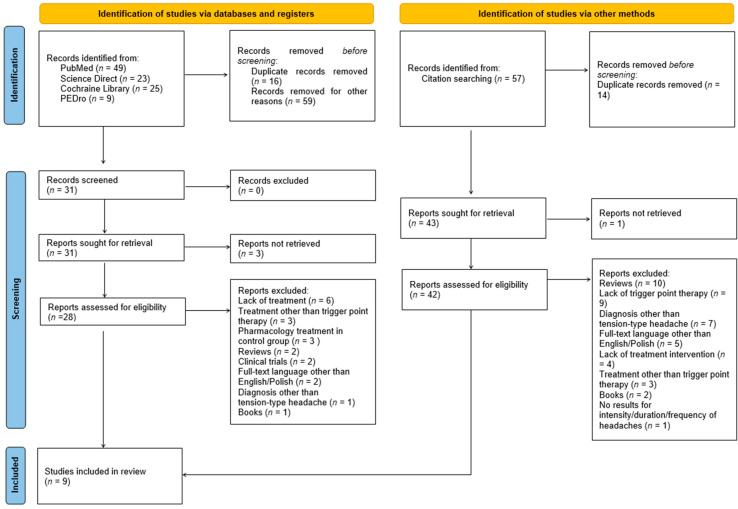
Flow diagram of study selection.

The initial database searches were conducted in May 2021 in four electronic databases: PubMed, Science Direct, Cochrane Library, and PEDro. The databases were selected based on previous systematic reviews [17,18,19,20]. The reference lists of all identified studies were manually examined to identify any articles missed by the electronic literature search. There were no restrictions on years of publication. The following formulas were used to search the databases: PubMed—“trigger point*” AND (“tension-type headache” OR “tension headache” OR “tension type headache”) AND (“physiotherapy” OR “physical therapy”); Cochraine Library -”trigger point*” AND (“tension-type headache” OR “tension headache” OR “tension type headache”) AND (“physiotherapy” OR “physical therapy” OR “therapy”); Science Direct—“trigger points” AND (“tension-type headache” OR “tension headache” OR “tension type headache”); PEDro—“trigger points” AND “tension-type headache”. The review process consisted of two stages. Three independent authors (A.D., M.B., W.W.) initially screened and identified relevant titles and abstracts. Then, full-text versions of articles were assessed against the inclusion and exclusion criteria. Any discrepancies regarding the inclusion of studies were resolved by the senior researcher (P.G.) [18]. Therefore, a total of 9 studies were included in the final analysis. The article selection procedure is presented in the flow diagram shown in Figure 1.

### 2.3. Data Extraction and Evaluation of the Methodological Quality of Studies

Two authors (W.W., M.B.) independently extracted data from the included articles and compared their findings for accuracy. The extracted date concerned the study design, participants (amount, TTH diagnosis, control group), treatment of TrP (treated muscles, type of TrP, trigger point therapy techniques), outcomes (general outcomes, pain intensity, frequency, duration, other), follow-up, and side effects.

The methodological quality of the included studies was evaluated by using the Physiotherapy Evidence Database (PEDro) Scale [21] and the Centre for Evidence-Based Medicine’s (CEBM’s) Levels of Evidence Scale [22]. Any disagreements on data extraction or evaluation of the methodological quality of studies were resolved by the third author (A.D.).

## 3. Results

Of the 9 included studies with 370 participants, 6 studies were randomised controlled trials, 2 were pilot studies, and 1 was a case report. The characteristics of the studies and their methodology can be found in Table 2. 

Pain intensity was the most frequently measured indicator of tension-type headache (nine of the nine studies included in the review). Seven out of the nine included studies [23,25,26,28,29,30,31] assessed the frequency of pain occurrence, and only four studies [25,29,30,31] measured the duration of a single episode of headache. Four studies assessed the quality of life [23,25,29,30]. Three studies assessed the pressure pain threshold (two local PPT [26,30], one local and distal PPT [28]). Two studies reported patients’ medication consumption [24,30]. Single studies assessed the range of cervical motion [26], the number of trigger points [24], the brain metabolite profile [28], and perceived clinical changes [30]. A follow-up was performed in five studies [23,25,27,29,30]. A summary of each study’s outcomes is presented in Table 3. 

The evaluation of the studies using the PEDro scale showed the diversified methodological quality of the included papers. The PEDro scores for the RCT ranged from 1 to 9. The work of Gildir et al. [25] received the highest PEDro score among the included studies (9/10 points—“excellent” quality). According to the PEDro scale, the studies of Kamali et al. [26], Abaschian et al. [23], Berggreen, Wiik, and Lund [24], Mohamadi et al. [28], and Moraska et al. [30] showed “good” quality of the methodology (between 6 and 8 points). The pilot studies of Moraska, Chandler [29], von Stülpnagel et al. [31], and the case study of Mohamadi, Ghanbari, and Rahimi Jaberi [27] were not considered in the PEDro scale analysis. The study of Gildir et al. [25] was also the only included paper rated 1b on the Levels of Evidence scale. The remaining experimental works were ranked as level 2b. The most common reason for a 2b rank was a small sample size and lack of follow-up. Based on the Levels of Evidence Scale, the pilot studies of Moraska, Chandler [29], von Stülpnagel et al. [31], and the case study of Mohamadi, Ghanbari, and Rahimi Jaberi [27] are ranked in place four. Full results of the study’s quality analysis are presented in Table 4. 

## 4. Discussion

Both clinical and scientific evidence suggests that the treatment of tension-type headaches should be based on pharmacological as well as non-pharmacological methods [32]. The previous research shows the validity of the unconventional, physiotherapeutic treatment of TTH [33,34]. One of the physiotherapy methods with proven effectiveness in TTH treatment is manual therapy, defined as a treatment consisting of a combination of mobilisations of the cervical and thoracic spine, exercises, and postural correction [13]. However, in the above-mentioned literature reviews, the myofascial trigger point therapy was not used to treat TTH or was part of the whole physiotherapy procedure. The knowledge about referred pain from trigger points as well as the involvement of trigger points in the peripheral and central sensitisation mechanisms suggests that the MTrP therapy may be of significant importance in unconventional TTH treatment. Therefore, the main aim of the literature review was to determine whether different trigger point therapy techniques are effective in decreasing of intensity, frequency, and duration of tension-type headaches.

In the studies included in the review, the most frequently used physiotherapeutic method was dry needling. According to The American Physical Therapy Association, DN is “a skilled intervention that uses a thin filiform needle to penetrate the skin and stimulate underlying myofascial trigger points, muscular, and connective tissues for the management of neuromusculoskeletal pain and movement impairments” [35]. The influence of DN on indicators of tension-type headaches was analysed in studies of Abaschian et al. [23], Gildir et al. [25], and Kamali et al. [26]. In the paper of Abaschian et al., the use of dry needling with passive muscle stretching was compared with passive stretching alone in people with episodic tension-type headaches. The inclusion of DN in the ETTH treatment protocol caused a significant improvement in the headache frequency and intensity. Moreover, performing only a passive stretch without inactivation of trigger points caused an increase in headache indicators. The authors of the study recommend the use of DN in the treatment of TTH due to the low costs and short duration of therapy [23]. In the study conducted by Gildir et al., the use of DN in patients with CTTH resulted in a reduction in the intensity, frequency, and duration of the pain episode, and the analgesic effect of DN was maintained after a monthly follow-up [25]. It is worth mentioning that in this study the control group consisted of patients who underwent sham-needling, which is a form of placebo. The lack of improvement in pain indicators in this group may suggest that the use of needle therapy in tension-type headaches may be particularly effective when it is aimed at deactivating trigger points. In the studies of Kamali et al., dry needling was as effective as friction massage. However, DN had a greater effect on increasing the pressure pain threshold of the muscles [26]. Only one study concerning dry needling in the treatment of TTH reported side effects. In the study by Gildir et al., the study participants reported pain and fear during needle application [25]. 

One of the most commonly used physiotherapeutic techniques to treat trigger points is ischaemic compression [36], which was used in the work of von Stülpnagel et al. [31] and Berggreen, Wiik, and Lund [24]. Ischaemic compression is defined as a manual technique in which the therapist applies pressure directly on the trigger point to reduce the blood supply and decrease the tension within the involved muscle [37,38]. In the Berggreen, Wiik, and Lund paper, ischaemic compression of trigger points reduced the intensity of the morning CTTH but did not affect the intensity of the pain in the evening. However, in this study, medicine consumption decreased significantly in the intervention group and increased in the control group, although not statistically significantly. The number of trigger points also decreased significantly in the intervention group. The authors suggest that due to the potential side effects of pharmacological treatment, myofascial trigger point massage should be considered an effective alternative in a clinical setting [24]. The treatment protocol of von Stülpnagel et al. included also local stretching of the taut band and active or passive stretching of the muscle combined with postisometric relaxation [31]. The results of this study show a reduction in all headache indicators—intensity, frequency, and duration. In both studies, the authors did not report any side effects [24,31]. 

Another technique for treating TrP in TTH was positional release techniques used in the paper of Mohamadi et al. [28] as well as Mohamadi, Ghanbari, and Rahimi Jaberi [27]. positional release techniques are osteopathic techniques aimed at increasing muscle flexibility by placing it in a shortened position to promote muscle relaxation [39]. Due to its delicate, passive character, PRT has been proposed as a technique for the treatment of chronic, subacute, and acute conditions [36]. These suggestions were confirmed by the studies included in the review. In the work of Mohamadi et al., the use of the positional relaxation technique reduced both the intensity and frequency of tension-type headaches [28]. The results of Mohamadi, Ghanbari, and Rahimi Jaberi’s studies suggested a reduction in the intensity of the patient’s tension-type headache, which lasted for the next 8 weeks until a family conflict occurred [27]. This result may be explained by the fact that psychological stress is one of the suggested factors in the development of trigger points [36]. 

In addition to using single techniques to treat tension-type headaches, two studies used a 45-min massage protocol focused on reducing the myofascial trigger point activity. In both studies, the use of a standardised massage protocol decreased headache frequency [29,30]. In the study of Moraska and Chandler, the intensity of the headache and duration of a single headache episode also decreased, both in patients with episodic and chronic headaches [29]. However, in the work of Moraska et al. these indicators have not changed in both the intervention, placebo, and waitlists group, and neither did medication use. Additionally, the frequency of headache decreased in both the intervention and placebo groups. The authors suggest that this finding may be explained by the fact that all chronic conditions are responsive to placebo [30]. However, it is worth mentioning that in this study only participants from the intervention group indicated a significant improvement in the perceived clinical change. Also, the pressure pain threshold increased only in the intervention group [30]. These discrepancies in the results of the study by Moraska et al. [30] indicate the need to implement a placebo group in research on pain caused by trigger points.

An additional aim of the literature review was to assess the impact of trigger point therapy on other physical and psychological variables in tension-type headaches. In addition to reducing the intensity, duration, and frequency of headaches, trigger point therapy appears to affect other aspects of pain in patients with tension-type headaches. The study by Mohamadi et al. [28] demonstrated a significant decrease in sensory, affective, and evaluative values, as well as in various aspects of pain measured by the Persian-language version of the McGill Pain Questionnaire. The research also confirmed the increase of the local pressure pain threshold in patients with tension-type headaches after the trigger point therapy [28]. The same result, i.e., an increase in local pain threshold, was achieved in a comparison study of dry needling and friction massage performed by Kamali et. al. [26]. In this study, DN was more effective in increasing the PPT compared to FM. Simultaneously, both methods did not affect the cervical range of motion. Differences were noticed only in extension, which increased in the dry needling group [26]. In opposition to the research of Mohamadi et al., the study results of Berggreen, Wiik, and Lund show no difference in the McGill Pain Questionnaire [24].

It is worth noting that some forms of tension-type headaches may be related to vascular abnormalities, and the therapies described may have a positive effect on treating them by promoting local vasodilation associated with these forms [40,41].

In addition to the influence on physiological variables, the MTrPs therapy may also affect other aspects of the TTH patient’s health. This has been confirmed by Gildir et al. who evaluated health-related quality of life factors by using a Turkish version of the Short Form-36 (SF-36) questionnaire in their study. In the dry-needling intervention group, a significant improvement was observed at the end of the therapy and during a 1-month follow-up in all factors of the Quality of Life test [25]. In contrast to these results, a study conducted by Abaschian et al., who also used the SF-36 questionnaire, obtained different results. Their study showed significant differences only in physical functioning and no significant changes affecting other aspects of quality of life. However, those results may arise from a lack of significant statistical differences between the quality of life in the groups before intervention [23]. The SF-36 questionnaire also showed no improvement in Berggreen, Wiik, and Lund’s research [24]. The increase in quality of life after TrP treatment was confirmed by the use of HDI and HIT-6 questionnaires in the study of Moraska et al. However, in this study, placebo therapy also contributed to the improvement of the quality of life measured with the HIT-6 scale [30]. The HDI questionnaire was also used in Morska and Chandler’s study on massage therapy, and it also showed improvement in the study subjects’ quality of life [29].

The aforementioned authors, who provide plausible evidence of the positive effect of the MTrP therapy on TTH, suggest as an explanation the phenomenon of referred pain, which is a pain felt in an often unexpected region, different from the original site of the painful stimulus [42]. Simons et al. describe referred pain as a repetitive pain from MTrP that does not follow myotomes, dermatomes, or nerve roots. MTrP pain occurs in the so-called maps (patterns) that indicate projection areas from specific myofascial trigger points [43]. Some cervical trigger points project pain into the head region and may be mistaken for headaches of other origins [44,45]. Fernandez-de-las-Penas et al. showed that tension-type headache patients had active myofascial trigger points evoking the same referred pain and sensory characteristics as their habitual headache [46,47]. Researchers have shown that several muscles, i.e., the upper trapezius, sternocleidomastoid, splenius capitis, or suboccipital muscles, can cause projected pain to the head mimicking a headache, which is simultaneously indicative of the role of active TrP [48,49]. Also, some previous studies have shown a direct relationship between the number of trigger points and tension-type headache indicators [50,51]. Another possible explanation of the positive effect of the TrP therapy on TTH is the phenomenon of central sensitisation (CS), defined as a neurophysiological mechanism that causes increased sensitivity and pain responses [52]. CS is associated with numerous chronic musculoskeletal conditions [53]. Previous research about the trigger point pain mechanisms suggests that MTrPs may play an important role in the sensitisation mechanism phenomenon. The clinical picture of pain may result from hyperalgesic and allodynic responses observed also in cases of TTH, which indicate the role of the peripheral and central mechanisms. Both hyper-excitability of the central nervous system, as well as reduction in the inhibitory mechanisms, are involved in tension-type headaches [50]. As a result of repeated or intense stimulation of the nociceptor present in the periphery, there may be an increase in excitability and the synaptic efficacy of the central nociceptive pathway neurons manifested as hypersensitivity to pain (tactile allodynia) [9,11]. In addition, the studies by Fernández-de-las-Peñas et al. revealed an elevated level of allogeneic substances in active TrP compared to the latent and tender points, which may be the cause for greater afferent stimulation into the nucleus caudalis. This could result in temporary and spatial summation of neuron signals and may lead to central sensitisation in chronic tension-type headaches [54]. It is suggested that central sensitisation in TTH is caused by the peripheral tissues generating prolonged peripheral nociceptive inputs, and it is dynamically influenced by activity and location of these nociceptive inputs [54]. This demonstrates that elimination of trigger points responsible for abnormal peripheral nociceptive stimulation may result in a reduction of synaptic activity of the central nociceptive pathway neurons, thereby reducing headache intensity. However, the results of this review do not provide a clear answer to the question of whether trigger point therapy in patients with TTH could affect central sensitisation. In the study of Kamali et al. [26], Mohamadi et al. [28], and Moraska et al. [30], the use of trigger point therapy resulted in an increase in the local pressure pain threshold. PPT is considered an adequate parameter in the research into central sensitisation [52]. In previous studies, injection of an anaesthetic into the trigger points of trapezius muscle resulted in a reduction in the peripheral pressure pain threshold in patients with neck pain after whiplash trauma [55]. Therefore, the results of the aforementioned studies may suggest that trigger point therapy may contribute to pain relief in TTH patients through the CS mechanism. In the Mohamadi et al. [28] studies, only local pressure pain threshold increased after trigger point therapy, while the distal PPT values did not change. In this study, also the brain metabolite profile was included in the assessment of central sensitisation. MRI examination showed no significant changes in any variables of the metabolite profile in the group with Positional Release Techniques treatment, which may suggest that this type of therapy did not affect the central nervous system. Thus, due to inconclusive results, the effect of trigger point therapy on central sensitisation in patients with tension-type headaches requires further research.

Results of most included studies have shown that trigger point therapy is effective in reducing pain indices in patients with tension-type headaches. The impact of TrP therapy on the improvement of the quality of life of patients with TTH is inconclusive. Treatment of trigger points increases the local pressure pain threshold. However, the lack of influence of TrP therapy on the distal PPT and brain metabolite profiles does not provide conclusive evidence that the central sensitisation mechanism is involved in the treatment of trigger points in patients with tension-type headaches. Nevertheless, the results of the PEDro assessment and the Levels of Evidence scale indicate the need to develop more refined test protocols, taking into account, e.g., blinded studies and larger study groups, to avoid discrepancies in the results of future research. Also, the inclusion of the group with placebo treatment is needed to not overestimate the positive effect of the trigger point therapy in chronic tension-type headaches.

## 5. Conclusions

- Trigger point therapy has reduced the duration, intensity, and frequency of headaches.
- Dry needling, ischaemic compression, positional relaxation techniques, and massage protocols focused on deactivating trigger points are effective methods of unconventional treatment of tension-type headaches.

## Figures and Tables

**Table 1:** Inclusion and exclusion criteria according to the PICO(s) strategy.

Studies	Participants	Interventions	Comparisons	Outcomes
Inclusion criteria				
All primary empirical study designs All years of publication English or Polish version of full-text Peer reviewed	Acute or chronic tension-type headaches No age restrictions	All physiotherapy techniques on TrP	Placebo group or other physiotherapy techniques or no comparator	Headache pain severity or frequency or duration or pressure pain threshold values
Exclusion criteria				
Reviews, editorials, letters to the editor, post-conference summaries, books Clinical trials Animal studies	Intervention groups involving other types of headache than tension-type Secondary headache	Physiotherapy treatment other than TrP therapy Non-physiotherapy treatment (eg. botulin injections) Acupuncture or acupressure treatment	Non-physiotherapy treatment	Studies that do not contain outcomes about headache pain severity or frequency or duration

**Table 2:** Characteristics and methodology of the included studies.

Reference	Type of Study	Participants, Diagnosis of TTH	Treatment	Control Group/Comparators	Type of TrP	Treated Muscles
Abaschian et al., 2020 [23]	RCT	*n* = 21 (investigation group—12 women, CG—9 women). ETTH was based on ICHD-3. criteria.	dry needling with passive stretching	passive stretch session only	active or latent	upper trapezius, sternocleidomastoid, temporalis
Berggreen et al., 2011 [24]	RCT	*n* = 35 (19—massage treatment; 16—CG); CTTH diagnosis was based on 1988 International Headache Society (IHS) criteria	ischaemic compression	no treatment	active	m. sternocleidomastoid, masseter muscle, m. temporalis, medial and lateral pterygoid muscles, anterior neck muscles, facial muscles, occipitofrontalis muscles, splenius capitis and splenius cervicis muscles, posterior cervical muscles, suboccipital muscles
Gildir et al., 2019 [25]	RCT	*n* = 160 (study group *n* = 80, control group *n* = 80); diagnosis of CTTH was based on the International Classification of Headache Disorders, 3rd edition beta version) (ICHD-3 beta criteria)	dry needling of active TrPs	placebo group—sham dry needling (into adipose tissue without TrPs)	active	upper trapezius, masseter, temporalis, frontalis, splenius cervicis and capitis, suboccipital
Kamali et al., 2019 [26]	RCT	*n* = 40 (20—friction massage group; 20—dry needling group); no information about TTH diagnosis criteria	2 groups—dry needling and friction massage	lack of CG—two different physiotherapy methods	active or latent	suboccipital, temporalis, SCM, and upper trapezius muscles
Mohamadi, Ghanbari, Rahimi Jaberi 2012 [27]	case report	*n* = 1; no information about TTH diagnosis criteria	Positional Release Therapy	lack of CG	active	right trapezius, left sternocleidomastoid, right and left obliqus capitis superior, left rectus capitis anterior
Mohamadi et al., 2020 [28]	RCT	*n* = 32 (SG—16, CG—16); diagnosis of TTH was based on the International Headache Society criteria (2004)	Positional Release Technique	lack of intervention	active	trapezius, sternocleidomastoid, obliquscapitis superior, rectus capitis anterior, rectus capitis posterior, interspinalis, and multifidus
Moraska, Chandler, 2008 [29]	pilot study	*n* = 16; (13—CTTH; 3—ETTH); the diagnosis of TTH was based on the International Headache Society criteria (2004)	massage focused on TrP	lack of CG	active	upper trapezius, sternocleidomastoid, suboccipital, and splenius capitis.
Moraska et al., 2015 [30]	RCT	*n* = 56 (17—massage group, 19—placebo group, 20—wait-list); TTH diagnosis was based on International Classification of Headache Disorders, 2nd edition (ICHD-2)	massage focused on TrP	placebo group- detuned ultrasound; wait-list group	active	upper trapezius, suboccipital muscles, sternocleidomastoid
von Stülpnagel et al., 2009 [31]	pilot study	*n* = 9; children; TTH diagnosis was based on the criteria of the International Headache Society	trigger point–specific physiotherapy—ischaemic compression, local stretching of the taut band, active and passive stretching of the muscle combined with postisometric relaxation.	lack of CG	active	sternocleidomastoid, splenius capitis, upper trapezius, temporalis, semispinalis, levator scapulae, masseter, and frontalis muscles

CG—control group; CTTH—chronic tension type headache; DN—dry needling; ETTH—episodic tension type headache; FM—friction massage; ICHD—The International Classification of Headache Disorders; MTrP-Myofascial Trigger Point; PRT—Positional Release Technique; RCT—randomised controlled trial; SCM—sternocleidomastoid muscle; SG-study group; TTH—tension-type headaches.

**Table 3:** Results of the included studies.

	Outcomes							Side Effects
Reference	Methods	General Outcomes	Pain Frequency	Pain Intensity	Pain Duration	Other	Follow-Up	
Abaschian et al., 2020 [23]	headache diary -intensity and frequency of headache; quality of life—SF-36; depression symptoms—Beck questionnaire	The intensity of tension-type headaches in the intervention group decreases statistically significantly after one month of intervention (*p* = 0.017). In the control group, the intensity of the headache increased after one month of intervention (*p* = 0.023). “One month after the intervention, there was a significant difference between the changes in headache intensity in the two groups (*p* = 0.003)”. Statistically, changes in headache frequency were significant after 4 weeks of intervention in the intervention group (*p* = 0.021), but they were not significant in the control group (*p* = 0.805). “There was a significant difference between the frequency changes of headache between two groups after 4 weeks of intervention (*p* = 0.018)”.	IG—baseline 11.08 (3.60), after 1 month—10.33 (3.67); *p* = 0.021 * CG—baseline 11.44 (3.77), after 1 month 11.77 (3.96) *p* = 0.805 [days per month]	IG—baseline 6.08 (1.23), after 1 month 5.52 (1.07); *p* = 0.017 * CG—baseline 4.83 (1.14), after 1 month 5.12 (1.08) *p* = 0.023 * [VAS 0–10]	ND	physical functioning— there was a significant difference between the control and intervention groups (*p* = 0.008) in terms of changes in physical functioning and quality of life. In the other areas of quality of life (role limitations, vitality, mental health, social health, bodily pain, genetal health), there was no significant difference between the two groups of intervention and control.	1 month after treatment	ND
			*p*-value between group = 0.018 *	*p*-value between group = 0.003 *				
Berggreen et al., 2011 [24]	headache diary—intensity (morning and evening pain, pain inconvenience and medicine consumption); McGill’s Pain Questionnaire (MPQ); number of TrPs—palpation; quality of life—SF-36	“The intention-to-treat analysis of the primary outcome (pain intensity in the morning), showed a significant decrease compared with the control group (difference 8.8 mm [95% CI 0.11—17.4], *p* = 0.047)”. “There were no significant differences between the treatment group and the control group for evening” (difference 3.4 mm [95% CI 9.3—16.0], *p* = 0.594).	ND	morning pain: TG—before treatment 28.0 (15.9), after treatment 16.2 (11.8), CG—before treatment 26.6 (12.6), after treatment 24.9 (14.5)	ND	VAS inconvenience morning and evening— no significant differences between the treatment group and the control group numer of TrPs— TG before treatment 37.9, after treatment 12.6; CG before treatment 43.2, after treatment 42.1, difference in the change: 31.2 [95% CI 20.8–41.5]) medicine consumption—TG before treatment 111.7 mg/day, after treatment 64.1 mg/day, CG before treatment 75.9 mg/day, after treatment 84.6 mg/day MPQ and SF-36—“There were no signifi cant differences between the treatment group and the controlgroup for MPQ pain score or SF-36 score”	-	ND
				*p*-value between group = 0.047 *				
				evening pain: TG—before treatment—34.7 (21.9), after treatment—22.2 (21.4), CG -before treatment 29.8 (18.2), after treatment 25.5 (16.3)				
				*p*-value between groups = 0.594				
Gildir et al., 2019 [25]	headache diary—TTH intensity, frequency and duration; quality of life—Turkish version of SF-36	“trigger point dry needling in patients with CTTH is effective and safe in reducing headache frequency, intensity and duration, and increasing health-related quality of life. Effectiveness of treatment begins in the first week of treatment and continues throughout the second week and follow-up periods” (1 month).	SG: before treatment 18.5 ± 2.7 after treatment 3.8 ± 1.8; CG: before treatment 18 ± 2.4 after treatment 7.9 ± 2.0 [day per month] *p*-value between group <0.05	SG: before treatment 4.5 ± 1.0 after treatment 0.7 ± 0.8; CG: before treatment 4.6 ± 1.2, after treatment 4.6 ± 0.7 [VAS 0–10 cm] *p*-value between group <0.05	SG: before treatment 3.9 ± 0.7, after treatment 0.7 ± 0.8; CG: before treatment 3.8 ± 0.9, after treatment 3.9 ± 1.0 [hours per day] *p* = 0.001	quality of life—Compared to the control group, the study group showed significantly lower results in all quality of life aspects (physical functioning, role physical, bodily pain, general health, vitality, social functioning, role emotional, mental health) after the treatment (*p* = 0.001).	after 4 weeks; pain intensity: SG 0.9 ± 0.9, CG 4.9 ± 0.7 [VAS 0–10 cm] pain frequency: SG 4.9 ± 2.8, CG 16.3 ± 2.6 [day per month]; pain duration: SG 0.7 ± 0.6, CG: 4.1 ± 0.8 [hours per day]	“Five of the patients in each group experienced pain and fear during the procedure”.
Kamali et al., 2019 [26]	headache frequency, headache intensity; pressure pain threshold–algometry; cervical range of motion—goniometer	“The results showed that both treatment methods significantly reduced headache frequency and intensity, and increased pain threshold at the trigger points” (*p* < 0.05). “Between-group comparisons showed that dry needling increased pain threshold significantly more than friction massage”.	DN group—baseline 5.00, after treatment1.95 ± 2.08, *p* < 0.05; FM group—7.00, after treatment 2.85 ± 2.56, *p* < 0.05 [days a week]	DN group—baseline 8.00, after treatment 3.00 ± 2.31, *p* < 0.05; FM group— baseline 9.50, after treatment4.22 ± 3.51, *p* < 0.05 [VAS 0–10]	ND	pressure pain treshold—DN group before treatment 1.07 (median), after treatment 1.27 ± 0.25 (mean ± SD), FM group before treatment 0.86 (median), after treatment 1.12 ± 0.42 (mean ± SD), between-group comparison after treatment—0.008 cervical range of motion	-	ND
			*p*-value between group = 0.7	*p*-value between group = 0.4				
Mohamadi, Ghanbari, Rahimi Jaberi 2012 [27]	Pain intensity—Numeric Pain Index (NPI)	“After 3 treatment sessions, the patient’s headache stopped completely. Throughout the next 8 months, she had no pain and did not use any medication. Unfortunately, after this time and following a family conflict, her headache returned”.	ND	before treatment—10; after first session—10; after second session—8; after third session—0 [NPI 0–10]	ND	ND	“Throughout the next 8 months, she had no pain and did not use any medication”.	ND
Mohamadi et al., 2020 [28]	headache diary—headache frequency and intensity; PPT—algometry; protone resonance spectroscopy; McGill Pain Questionnaire	Headache frequency (*p* = 0.001) and intensity (*p* = 0.002) decreased significantly in the PRT group after treatment.	SG: before treatment 18.30 ± 6.29, after treatment 5.84 ± 3.76, *p* = 0.001 * CG: before treatment 16.69 ± 6.14, after treatment 17.30 ± 6.57, *p* = 0.36 [days per month]	SG: before treatment 7.46 ± 1.80, after treatment 4.38 ± 1.66, *p* = 0.002 * CG: before treatment 6.53 ± 1.89, after treatment 7.00 ± 1.47, *p* = 0.14 [0–10]	ND	metabolite profile—SG: no significant changes after treatment, CG: no significant changes in any variables except M-Ino/Cr ratio in the somatosensory cortex, which increased significantly (*p* = 0.041). McGill score—McGill score (*p* = 0.003) decreased significantly in the PRT group aftertreatment. Statistically significant differences between groups were found (F_1,22_ = 24.02; *p* < 0.001; eta = 0.52) local PPT: SG before treatment 1.99 ± 0.55, after treatment 2.60 ± 0.74 (*p* = 0.003), CG before treatment 2.34 ± 0.84, after treatment 2.33 ± 0.81 (*p* = 0.88). distal PPT: SG before treatment 6.10 ± 1.63, after treatment 6.53 ± 1.36 (*p* = 0.23), CG before treatment 5.01 ± 1.49, after treatment 4.79 ± 1.66 (*p* = 0.19). Statistically significant differences between groups were found for local PPT (F_1,22_ = 10.31, *p* = 0.004, eta = 0.32) and distal PPT (F_1,22_ = 4.72, *p* = 0.04, eta = 0.17).	-	ND
			*p*-value between group < 0.001 *	*p*-value between group < 0.001 *				
Moraska, Chandler, 2008 [29]	headache diary—frequency (primary outcome) and peak intensity, duration (secondary outcome)	“Headache frequency decreased from 4.7 ± 0.7 episodes per week during baseline to 3.7 ± 0.9 during treatment period 2 (*p* < 0.05); reduction was also noted during the follow-up phase (3.2 ± 1.0). Secondary measures of headache also decreased across the study phases with headache intensity decreasing by 30% (*p* < 0.01) and headache duration from 4.0 ± 1.3 to 2.8 ± 0.5 h (*p* < 0.05)”.	CTTH group—baseline: 5.33 ± 0.54, 3 weeks 4.93 ± 0.75; 6 weeks 4.28 ± 0.88; follow up 3.67 ± 1.18 ETTH group- baseline 2.57 ± 0.85; 3 weeks 2.57 ± 1.71; 6 weeks 1.00 ± 0.33; follow-up 1.33 ± 0.33 [days per week] *p* < 0.01	CTTH group—baseline 43.2 ± 6.30; 3 weeks 38.7 ± 9.18; 6 weeks 31.9 ± 9.39; follow-up 32.8 ± 10.3 ETTH group—baseline 60.3 ± 22.5; 3 weeks 41.2 ± 20.0; 6 weeks 43.1 ± 28.4; follow-up 40.6 ± 23.2 [VAS 0–100 mm] *p* = 0.001	CTTH group—baseline 3.58 ± 1.10; 3 weeks 3.96 ± 1.01; 6 weeks 2.85 ± 0.70; follow-up 2.70 ± 0.48 ETTH group—baseline 5.73 ± 5.38; 3 weeks 4.77 ± 3.80; 6 weeks 5.37 ± 3.93; follow-up 2.90 ± 1.65 [hours] *p* < 0.05	Headache Disability Index questionnaire: HDI- Total: CTTH group—baseline: 43.6 ± 7.66, 3 weeks 34.0 ± 9.55; 6 weeks 23.3 ± 10.6; follow up 26.4 ± 9.20 ETTH group—baseline 47.0 ± 29.1; 3 weeks 40.6 ± 34.6; 6 weeks 33.4 ± 14.6; follow-up 26.6 ± 10.2 HDI- Emotional: CTTH group—baseline: 17.8 ± 3.58, 3 weeks 14.3 ± 4.19; 6 weeks 9.08 ± 4.32; follow up 9.85 ± 4.18 ETTH group—baseline 19.0 ± 13.1; 3 weeks 15.3 ± 12.9; 6 weeks 10.7 ± 7.28; follow-up 7.33 ± 3.46 HDI—Functional: CTTH group—baseline: 25.8 ± 4.80, 3 weeks 19.7 ± 5.66; 6 weeks 14.2 ± 6.57; follow up 16.5 ± 5.34 ETTH group—baseline 28.0 ± 16.1; 3 weeks 25.3 ± 21.8; 6 weeks 22.7 ± 7.96; follow-up 19.3 ± 6.92	after 3 weeks	ND
Moraska et al., 2015 [30]	headache diary—frequency, intensity, and duration, medication use (dose/wk); PPT—algometry; quality of life—Headache Disability Inventory, Headache Impact Test (HIT-6); perceived clinical change	“…group differences across time were detected in HA frequency (*p* = 0.026), but not for intensity or duration. Post hoc analysis indicated that HA frequency decreased from baseline for both massage (*p* < 0.0003) and placebo (*p* = 0.013), but no difference was detected between massage and placebo. Patient report of perceived clinical change was greater reduction in HA pain for massage than placebo or wait-list groups (*p* = 0.002)”.	Massage group—baseline 3.72 ± 0.23, after 3 weeks of treatment 3.38± 0.31, after 6 weeks of treatment 2.37 ± 0.36, *p* = 0.0003 *; Placebo group—baseline 3.81 ± 0.21, after 3 weeks of treatment 3.21 ± 0.29, after 6 weeks of treatment 2.92 ± 0.34, *p* = 0.013 *; Wait-list group—baseline 3.69 ± 0.21, after 3 weeks of treatment 3.54 ± 0.29, after 6 weeks of treatment 3.67 ± 0.33, *p* = 0.098. *p*-value between massage group and placebo group = 0.26 Group × Time Interaction = 0.026 *	Massage group—baseline 31.4 ± 2.69, after 3 weeks of treatment 26.3 ± 2.50, after 6 weeks of treatment 27.3 ± 3.04; Placebo group—baseline 33.3 ± 2.52, after 3 weeks of treatment 30.8 ± 2.34, after 6 weeks of treatment 29.8 ± 2.82; Wait-list group—baseline 31.2 ± 2.46, after 3 weeks of treatment 27.8 ± 2.28, after 6 weeks of treatment 29.5 ± 2.75. “No significant treatment group differences weredetected for HA intensity” Group × Time Interaction = 0.03	Massage group—baseline 3.15 ± 0.43, after 3 weeks of treatment 3.20 ± 0.55, after 6 weeks of treatment 2.81 ± 0.50; Placebo group—baseline 2.86 ± 0.40, after 3 weeks of treatment 2.70 ± 0.52, after 6 weeks of treatment 2.84 ± 0.46; Wait-list group—baseline 3.02 ± 0.39, after 3 weeks of treatment 3.53 ± 0.51, after 6 weeks of treatment 3.36 ± 0.45 [hours]. “No significant treatment group differences were detected for HA duration” Group × Time Interaction = 0.49	PPT—There was a significant time by treatment interaction for all the 4 sites tested (upper trapezius, left and right and suboccipital, left and right) (F values ranged from 4.49 to 7.91, *p* values ranged from <0.001 to 0.015). Post hoc analyses showed that scores ignificantly improved in the massage group (*p* values ranged from <0.001 to 0.002 across outcomes), but did not change in the placebo and wait-list groups (all Ps > 0.17). Quality of life—Post hoc tests showed a significant decrease in HDI scores in the intervention group (*p* = 0.0003) but not in the placebo (*p* = 0.06) or wait-list (*p* = 0.39) groups. A significant change in HIT-6 scores was detected over time in both the intervention (*p* = 0.0002) and placebo (*p* = 0.011) groups but not in the wait-list group (*p* = 0.52). perceived clinical change—end of treatment: massage group—small negative change 7.7%, no change 0%, small positive change 7.7%, moderate positive change 46.2%, large positive change 38.5%; placebo—small negative change 5.6%, no change 16.7%, small positive change 27.8%, moderate positive change 33.3%, large positive change 16.7%; wait-list—small negative change 5.9%, no change 82.4%, small positive change 11.7.%, moderate positive change 0%, large positive change 0%, *p* < 0.0001. perceived clinical change—after follow-up: massage group—small negative change 7.1%, no change 7.1%, small positive change 21.4%, moderate positive change 28.6%, large positive change 35.7%; placebo—small negative change 0%, no change 42.1%, small positive change 21.0%, moderate positive change 21.0%, large positive change 15.8%; wait-list—small negative change 0%, no change 81.2%, small positive change 12.5%, moderate positive change 6.2%, large positive change 0%, *p* = 0.002.	4 weeks after end of treatment; pain intensity: massage group 22.8 ± 3.03, placebo group 31.5 ± 2.80, wait-list group 29.0 ± 2.74 [VAS 0–100]; pain frequency: massage group 2.61 ± 0.35, placebo group 2.96 ± 0.33, wait-list group 3.19 ± 0.32 [days per week]; pain duration: massage group 2.65 ± 0.4, placebo group—3.01 ± 0.44; wait-list group 3.12 ± 0.43 [hours].	
von Stülpnagel et al., 2009 [31]	headache diary—frequency, intensity, duration	“The headache frequency had been reduced from more than 3 days per week to 1 day per week (67.7% improvement). The intensity had nearly diminished to 1.67 on the visual analog scale compared to 6.5 visual analog scale before treatment (74.3% improvement). The headache duration also improved significantly from 6 h/d before treatment to 1.36 h/d after treatment (77.3% improvement)”.	before treatment 3.1 (1.5–7); after treatment 1 (0–3) [day per week] 67.7% improvement	before treatment 6.5 (5.5–8.5); after treatment 1.67 (0–4) [VAS 0–10] 74.3% improvement	before treatment 6 (1.5–12); after treatment 1.36 (0–2.5) [hours per day] 77.3% improvement	ND	-	no side effects

CG—control group; CTTH—chronic tension type headache; DN—dry needling; ETTH—episodic tension type headache; FM—friction massage; HA—headache; HDI—Headache Disability Index; HIT—headache impact test; IG—intervention group; MPQ—McGil’s Pain Questionnaire; ND—no data; NPI—numeric pain index; PPT—pressure pain threshold; PRT—positional release technique; SF-36—Short Form-36; SG—study group; TG—treatment group; TTH—tension type headache; VAS—visual analogue scale; *—statistical significance

**Table 4:** Analysis of the methodological quality of included studies according to the Physiotherapy Evidence Database (PEDro) Scale and the Centre for Evidence-Based Medicine’s (CEBM’s) Levels of Evidence Scale.

	Abaschian et al., 2020 [23]	Berggreen, Wiik and Lund, 2011 [24]	Gildir et al., 2019 [25]	Kamali et al., 2019 [26]	Mohamadi, Ghanbari, Rahimi Jaberi 2012 [27]	Mohamadi et al., 2020 [28]	Moraska, Chandler, 2008 [29]	Moraska et al., 2015 [30]	von Stülpnagel et al., 2009 [31]
Eligibility criteria were specified (without points)	Y	Y	Y	N	-	Y	-	Y	-
Subjects were randomly allocated to groups	1	1	1	1	-	1	-	1	-
Allocation was concealed	1	0	1	1	-	1	-	1	-
The groups were similar at baseline regarding the most important prognostic indicators	0	1	1	1	-	1	-	1	-
There was blinding of all subjects	1	0	1	1	-	0	-	0	-
There was blinding of all therapists who administered the therapy	0	0	1	0	-	0	-	0	-
There was blinding of all assessors who measured at least one key outcome	0	0	1	0	-	0	-	0	-
Measures of at least one key outcome were obtained from more than 85% of the subjects initially allocated to groups	1	1	1	1	-	0	-	1	-
All subjects for whom outcome measures were available received the treatment or control condition as allocated	0	1	0	0	-	1	-	0	-
The result of between-group comparisons are reported for at least one key outcome	1	1	1	1	-	1	-	1	-
The study provides both point measures and measures of variability for at least one key outcome	1	1	1	1	-	1	-	1	-
**Total PEDro score**	**6**	**6**	**9**	**7**	**-**	**6**	-	**6**	**-**
**Level of Evidence**	**2b**	**2b**	**1b**	**2b**	**4**	**2b**	4	**2b**	**4**

**N—No; Y—Yes; -—Article Not Rated**.
