# Correction: Dolina et al. Trigger Point Therapy Techniques as an Effective Unconventional Method of Treating Tension Headaches: A Systematic Review. *Healthcare* 2024, *12*, 1868

**DOI:** 10.3390/healthcare12202079

**Published:** 2024-10-18

**Authors:** Aleksandra Dolina, Michał Baszczowski, Wiktor Wilkowicz, Grzegorz Zieliński, Jacek Szkutnik, Piotr Gawda

**Affiliations:** 1 Interdisciplinary Scientific Group of Sports Medicine, Department of Sports Medicine, Medical University of Lublin, 20-093 Lublin, Poland; 2 Department of Sports Medicine, Medical University of Lublin, 20-093 Lublin, Poland; 3 Independent Unit of Functional Masticatory Disorders, Medical University of Lublin, 20-093 Lublin, Poland

In the original publication [1], there was a mistake in Figure 1 as published. On the left side of the figure, at the screening stage “reports sought for retrieval”, instead of the number *n* = 3, there should be *n* = 31, while, on the right side of the figure, at the screening stage “reports sought for retrieval”, instead of the number *n* = 1, there should be *n* = 43. The corrected Figure 1 appears below. The authors state that the scientific conclusions are unaffected. This correction was approved by the Academic Editor. The original publication has also been updated.

## Figures and Tables

**Figure 1:**
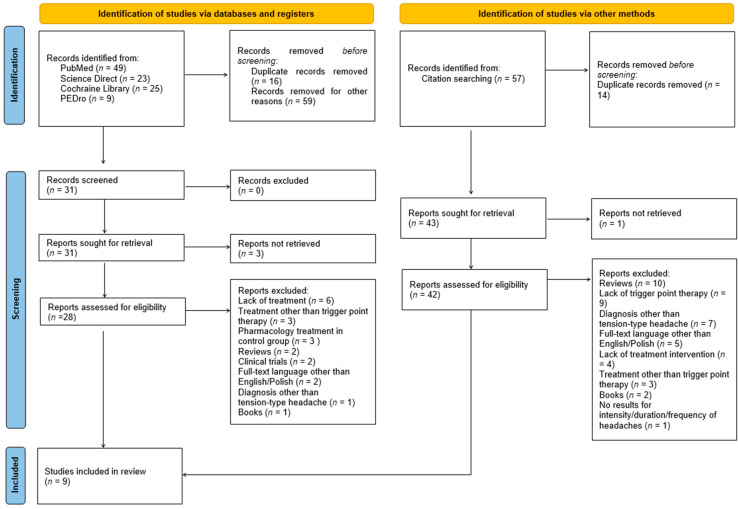
Flow diagram of study selection.
